# Crosslinked All-Femtosecond Laser-Cut Corneal Allogenic Intracorneal Ring Segments (AFXL CAIRSs): Pilot Ex Vivo Study and Report of First Two Cases Performed in Italy

**DOI:** 10.3390/jcm13195771

**Published:** 2024-09-27

**Authors:** Cosimo Mazzotta, Marco Zagari, Giulia Bona, Diego Ponzin, Shady T. Awwad, Emilio A. Torres-Netto, Farhad Hafezi, Soosan Jacob

**Affiliations:** 1 Department of Medicine, Surgery and Neurosciences, Postgraduate Ophthalmology School, Siena University, 53035 Siena, Italy; 2 Departmental Ophthalmology Unit, AUSL Toscana Sud Est, 53035 Siena, Italy; 3 Siena Crosslinking Center, 53035 Siena, Italy; 4 Vampolieri Eye Clinic, 95021 Castello, Italy; marco.zagari@hotmail.com (M.Z.); giuliabona91@hotmail.it (G.B.); 5 Veneto Eye Bank Foundation, 30174 Venice, Italy; diego.ponzin@fbov.it; 6 Department of Ophthalmology, American University of Beirut-Medical Center, Beirut 1107-2020, Lebanon; shadyawwad@yahoo.com; 7 ELZA Institute, 8953 Dietikon, Switzerland; etorres@elza-institute.com (E.A.T.-N.); fhafezi@elza-institute.com (F.H.); 8 Ocular Cell Biology Laboratory, University of Zurich, 8001 Zurich, Switzerland; 9 Department of Cornea, Dr. Agarwal’s Eye Hospital and Eye Research Centre, Chennai 600018, India; dr_soosanj@hotmail.com; 10 Department of Cornea, Dr. Agarwal’s Refractive and Cornea Foundation, Chennai 600006, India

**Keywords:** crosslinked CAIRS, crosslinked all-femtosecond laser-cut CAIRS, keratoconus, asymmetric CAIRS, AFXL CAIRS, corneal allogenic intrastromal rings, crosslinked allogenic intrastromal corneal rings, crosslinked ICRS

## Abstract

**Objectives:** This pilot ex vivo study and first clinical experience in Italy evaluate the impact of using pre-implantation crosslinking on all-femtosecond laser-cut corneal allogenic intracorneal ring segments (AFXL CAIRSs). **Methods:** Six human donor eye-bank corneas were used for this preclinical ex vivo human study. Three donor (D) corneas were used for AFXL CAIRSs. First, they were prepared with an IntraLase™ femtosecond laser (Johnson & Johnson, New Brunswick, NJ, USA). The allogenic tissue rings were crosslinked before implantation with Riboflavin–UV-A accelerated crosslinking protocol (ACXL) with a 0.1% HPMC Riboflavin isotonic solution (Vibex Rapid, Glaukos-Avedro, Burlington, MA, USA) and a new KXL UV-A emitter (Glaukos-Avedro, USA). Three corneas were used as recipients (Rs) of the AFXL CAIRSs. After completing the ex vivo phase, IRB approval and signing a specific informed consent, the first two Italian patients were treated. A single ACXL CAIRS was implanted in a 51-year-old male with 53.53 D K steep, 363 μm minimum corneal thickness (MCT) and a double ACXL CAIRS was implanted in a 46-year-old male patient with 58.30 D K steep, 443 μm MCT. The longest follow-up was at three months. **Results:** Crosslinking of the segments enhanced tissue stiffness and grip, facilitating manipulation and CAIRS insertion into the recipient tunnels, and the yellowish color of the crosslinked segments improved visibility. The segment’s thickness and volume remained unaltered during the follow-up. Both patients improved UDVA and BSCVA. K steep and High-Order Aberrations (HOAs) were reduced and MCT increased. **Conclusions:** Pre-implantation ACXL facilitated CAIRS insertion preserving dimensions and volume during the follow-up, rendering this important step a promising candidate in method standardization. Functional data and MCT improved significantly without adverse events.

## 1. Introduction

Corneal allogenic intrastromal ring segments (CAIRSs) were introduced by Soosan Jacob [1,2] in 2015 as an alternative to intracorneal synthetic ring segment implantation for keratoconus (KC) patients, aiming to reinforce and reshape the corneal surface, reduce High-Order Aberrations, and improve visual acuity [1,2]. This therapy involves the intrastromal implant of allogenic donor cornea arcuate stromal segments (of different shapes and angular degrees of thickness) into the mid-peripheral intrastromal layer through manual or femtosecond laser-assisted methods. CAIRSs avoid adverse events common with plastic intrastromal corneal ring segments (ICRSs) such as migration, exposure, stromal melt, necrosis, extrusion, neovascularization, infectious keratitis, etc. [3,4].

Research indicates that CAIRSs allow the passage of oxygen and nutrients, unlike synthetic segments [5,6], promoting better tolerance in the recipient cornea as these are made of allogenic tissue such as donor stroma, which can be easily integrated into the host tissue. Being allogenic and less rigid, CAIRSs can be suitable for more advanced KC cases with thinner corneas (between 300 and 400 µm) as they do not require a minimum thickness of 400 µm to prevent stromal necrosis and melt [1]. CAIRSs offer more customizability [2,5] in terms of arc length, thickness, optical zone and depth of implantation. Additionally, CAIRS recipients experience less glare compared to patients with synthetic segments due to the absence of reflections [6].

In order to flatten the learning curve, particularly segment insertion, different methods have been reported beyond femtosecond laser-assisted preparation, such as varying extents of dehydration and trypan blue staining for intraoperative ring visualization [7,8,9,10]. Corneal collagen crosslinking (CXL) has been used for 20 years to stop or delay corneal ectasia progression by inducing stromal stiffening [11]. CXL also enhances resistance to stromal enzymatic digestion by inhibiting collagenase activity and [12,13,14]. Moreover PACK-CXL is now more frequently used to treat infections from various infectious origins [15].

This study presents the first pilot ex vivo study and first in vivo clinical application of AFXL CAIRSs (crosslinked all-femtosecond laser-cut corneal allogenic intracorneal ring segments) using donor human corneas. Corneas unsuitable for corneal transplant due to low endothelial cell count and polymorphism were used for the preclinical ex vivo human study, while CAIRS preparation was performed in corneas suitable for lamellar keratoplasty. All corneas were provided by the Veneto Eye-Bank Foundation, Mestre, Italy. The aim of the preliminary ex vivo study was to determine if pre-implantation Riboflavin–UVA-induced crosslinking enhances the surgical technique by increasing corneal rigidity, maintaining its shape and thickness and thus facilitating CAIRS insertion into recipient tunnels. The aim of the clinical study was to verify the usefulness of crosslinking in facilitating the all-femtosecond laser-cut CAIRS surgical technique and evaluating the tomographic and functional changes induced by the implantation of crosslinked CAIRSs in two keratoconic patients intolerant to rigid gas permeable contact lenses and with poor best spectacle-corrected visual acuity.

## 2. Methods

### 2.1. Phase 1: Ex Vivo Preclinical Study

**Figure 1:**
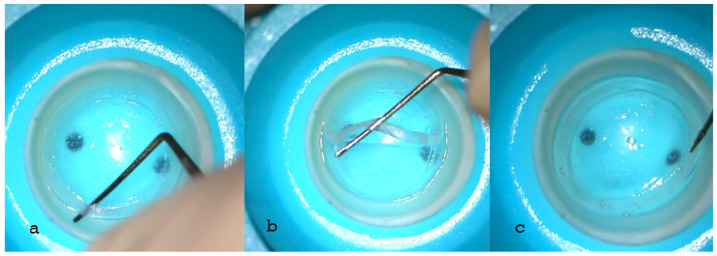
IntraLase™ (Johnson & Johnson, NJ, USA) all-femtosecond laser-cut CAIRS extraction from the donor cornea using a blunt spatula (**a**–**c**). A raster cut of 9 mm was planned to an adequate depth in the donor tissue in relation to the thickness of the full 360° ring to be constructed and based on the tomographic characteristics of the recipient cornea. This was followed by all-femtosecond laser-cut double spiral cuts, using 1.5 micro-Joules (μJ) energy at 6.5 and 8 mm in diameters, respectively, with different angles of inclination. In all cases, 1.5 mm wide, 300 µm thick (without epithelium), 160° arch length allogenic rings were prepared after full-ring extraction.

After joint approval of the pilot study protocol by the Siena Crosslinking Center, Siena, and Vampolieri Eye Clinic, Acicastello, Catania, Italy, Institutional Review Boards (IRBs), six human donor eye-bank corneas, unsuitable for corneal transplant due to low endothelial cell count and polymorphism, were used for the preclinical ex vivo human study. The corneas were mounted on a single-use testing anterior chamber (Moria, Bourbon, France) and subjected to tomographic analysis with an MS 39 OCT Tomographer (CSO, Florence, Italy) to evaluate total, epithelial and stromal minimum corneal thickness, anterior and posterior curvatures, and aberrations. Three donor corneas (D1, D2, D3) were used for AFXL CAIRSs, cut and prepared with an IntraLase™ femtosecond laser (Johnson & Johnson, New Brunswick, NJ, USA) and a software the authors (CM, MZ) specifically modified and developed. A raster cut of 9 mm was performed to an adequate depth based on the tomographic characteristics of the recipient cornea, relative to the thickness of the ring to be constructed. This was followed by double spiral cuts at diameters of 6.5 and 8 mm, respectively, to obtain a 1.5 mm wide, 300 µm thick (without epithelium), 160° arch length allogenic ring, and specific external and internal inclination angles for trapezoid shape customization were utilized, as shown in Table 1 and Figure 1.

Preparation of the donor ring was performed from the epithelial side to preserve the integrity of Bowman’s lamina. The epithelium was manually removed by a blunt metal spatula. The allogenic tissue rings were immediately crosslinked before implantation using the Riboflavin–UV-A accelerated crosslinking (ACXL) protocol [10], employing a 0.1% HPMC Riboflavin isotonic solution (Vibex Rapid, Glaukos-Avedro, Burlington, MA, USA) and a new KXL UV-A emitter (Glaukos-Avedro, Burlington, USA) [10]. All the CAIRSs were imbibed by immersion in a Riboflavin 0.1% plus HPMC 1% bath for 5 min and, after BSS rinsing (10 s), irradiated for 3, 5 and 10 min with an irradiance of 9 mW/5.4 J/cm^2^.

Three donor corneas were used as recipients (R1, R2, R3) of the AFXL CAIRSs. The femtosecond laser created 2 mm diameter and 160° arc length tunnels in the recipients for all corneas. Implants were centered on the corneal vertex and the optical zones were planned at 6.0 mm to ensure that the treatment encompassed the scotopic pupil diameter. The depth of the ACXL CAIRS implants varied from 50% to 70% to verify the feasibility of ring insertion and the variations in corneal curvature of the recipient corneas. Beyond the all-femtosecond laser-cut segment creation, the main objective of the pilot study was to evaluate the effect of pre-insertion crosslinking on the allogenic corneal segments in terms of increased resistance, viscoelastic consistency, ease of insertion into the recipient tunnels and maintaining the planned shape and volume for the purpose of taking a further step towards future surgical technique standardization.

**Figure 2:**
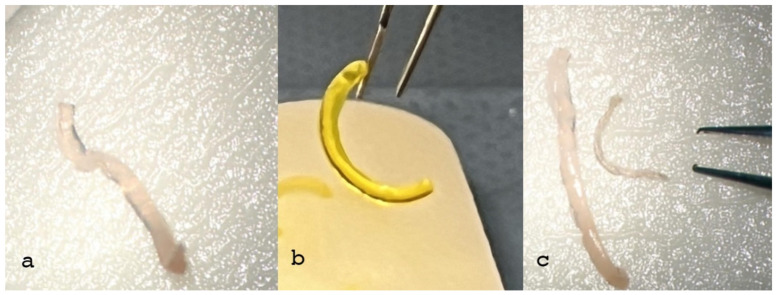
Non-crosslinked CAIRS (**a**) demonstrates softness and deformability, resulting in poor consistency, grip and visibility. In contrast, the crosslinked allogenic ring (**b**) shows better consistency, shape retention (trapezoid in shape, 1.5 mm wide, 300 µm thick) and significantly improved surgical grip for insertion maneuvers. Although ring dehydration (**c**) increases consistency it reduces ring volume, altering the original shape, and it does not improve visibility, which consequently requires additional coloring, potentially resulting in poorer results.

The three corneas with the greatest thicknesses were chosen as donors (D1, D2, D3) for the preparation of the 300 µm (without epithelium), 1.5 mm wide, 160° arch length CAIRSs, while the three thinnest corneas were designated for implantation as recipients. Coincidentally, tomographic analysis revealed the presence of a keratoconus in the thinnest donor cornea. The stiffening effect of ACXL and the Riboflavin-induced yellowish coloration rendered the allogenic ring stronger, less deformable and more malleable, improving grip and visibility, as shown in Figure 2b, compared to the non-crosslinked, soft and dehydrated segments (Figure 2a,c).

**Figure 3:**
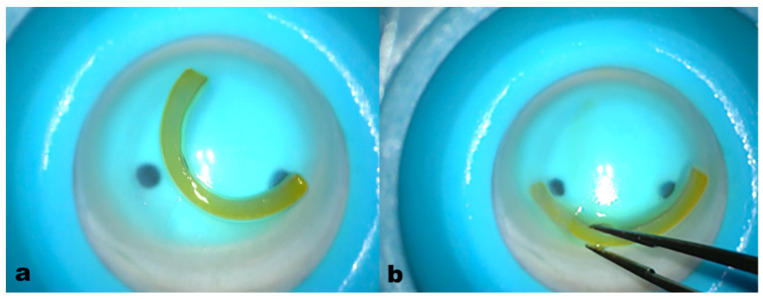
All-femtosecond laser-cut ACXL CAIRS before insertion (**a**) and during the insertion maneuver (**b**). The crosslinking of the segments produced stronger tissue with better grip and visibility, facilitating more precise and easier manipulation and insertion into the 2 mm diameter recipient tunnels. This significantly improved the maneuver without altering the thickness and volume of the segment (1.5 mm wide, 300 µm thick) and the yellowish Riboflavin-induced coloration enhanced AFXL CAIRS visibility without needing additional coloring.

The addition of crosslinking improved the rigidity and the viscoelastic consistency of the corneal allogenic rings while maintaining the dimensions and volume of CAIRSs (1.5 mm width, 300 µm thickness without epithelium, 160° arch length). This facilitated insertion into the recipient’s tunnels and consequently flattened the learning curve of the technique, Figure 3.

### 2.2. Phase 1 Ex Vivo Preclinical Study Outcomes

**Figure 4:**
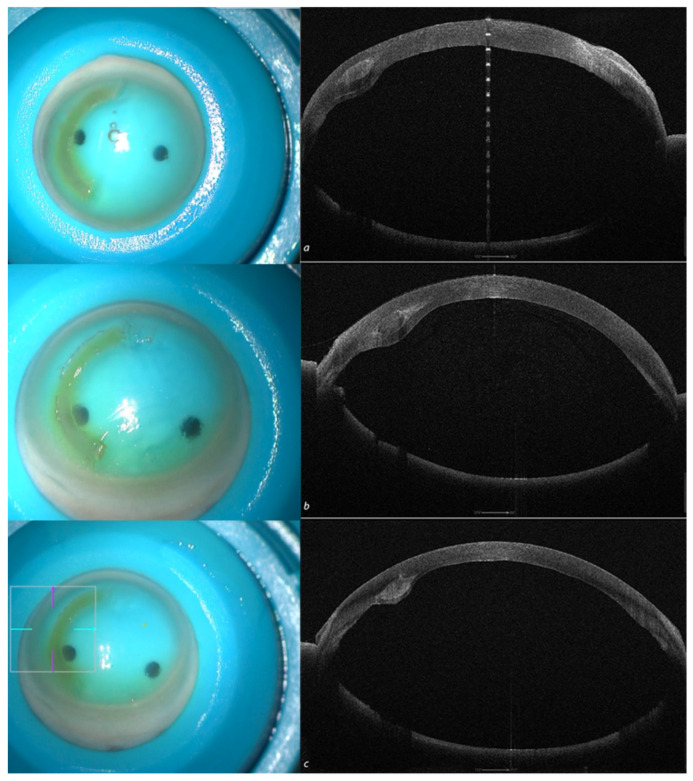
Recipient corneas with crosslinked all-femtosecond laser-cut CAIRS (AFXL CAIRS) implants at different depths, well-visualized by high-resolution (HR) anterior segment OCT tomography performed with an MS 39 (CSO, Florence, Italy) at 50% (**a**), 60% (**b**) and 70% (**c**).

The test was successful, independent of the implant depth (50, 60 and 70%), as shown in Figure 4.

The crosslinked ring maintained its shape and volume (trapezoid, 1.5 mm wide, 300 µm thick) with no observed differences pre- and post-CXL, as shown in Figure 2b. In contrast, the non-crosslinked ring was soft, bent easily and lost its original volume and shape, as shown in Figure 2a,c.

Crosslinking the segments enhanced manipulation and accurate insertion into the tunnels, significantly improving the surgical maneuver. The process did not alter the thickness and volume of the segment compared to the non-crosslinked, dehydrated segment. Additionally, the yellowish coloration of the crosslinked segment facilitated visibility and positioning (Figure 3).

**Figure 5:**
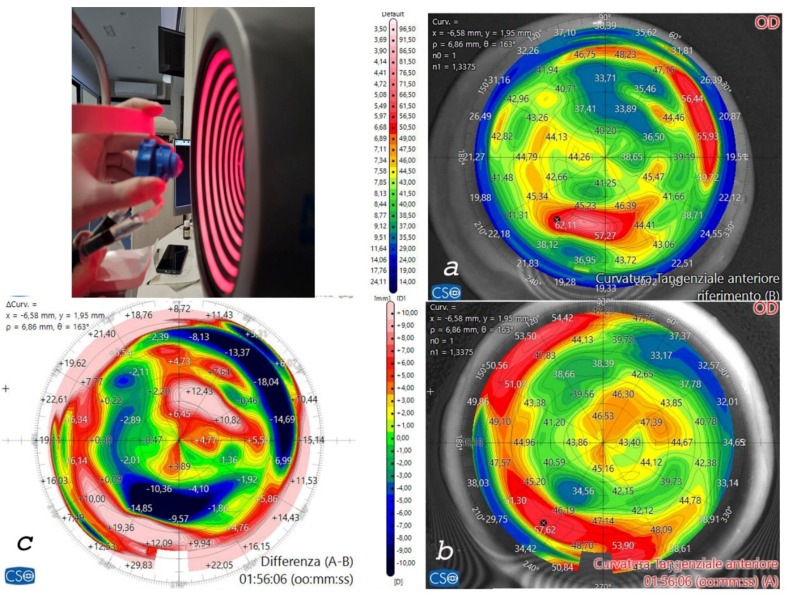
Human donor corneas were mounted on a single-use testing anterior chamber and subjected to tomographic analysis with an MS 39 OCT Tomographer (CSO, Florence, Italy), as shown in the bottom left image. Incidentally, the tomographic analysis revealed the presence of a keratoconus (**a**) in one of the recipient corneas and the implantation of the crosslinked allogenic ring allowed for reshaping of the corneal curvature, as documented by the postoperative scan (**b**) and differential tangential map (**c**).

The anterior testing chamber allowed for pre- and postoperative tomographic analysis of all corneas. This enabled us to select the three thickest samples for the preparation of the 300 µm (without epithelium), 160° arch length trapezoid rings and the three thinnest corneas to receive the implants. Furthermore, the tomographic analysis facilitated decisions on ring thickness, internal and external angle cut inclinations, and recipient cornea implantation depth, as shown in Figure 5. The recipient corneas with the thinnest thickness and with keratoconus (Figure 5a) showed corneal profile regularization after ACXL CAIRS implantation, as can be seen from the postoperative scan and differential tangential map (Figure 5b).

The ex vivo changes in corneal thickness, maximum corneal curvature (K max) and High-Order Aberration (HOA) values following ACXL CAIRS implantation are reported in Table 2.

The overall data on corneal thickness (both central and at the implant site) and High-Order Aberration (HOA) demonstrate the high potential of this additive technique in keratoconus in the preclinical ex vivo study. It induces a centripetal tissue shift, similar to a tissue wave, capable of moving over 13 diopters of curvature with a standard deviation of 5.62 D, as shown in Table 3.

### 2.3. Phase 2: Pilot Clinical Study in Italy

#### 2.3.1. Patient 1: Single ACXL CAIRS Implant

A 51-year-old male intolerant to rigid gas permeable contact lens (RGL CL) with stage II duck-shape asymmetric KC, who had undergone a deep anterior lamellar keratoplasty in the left eye, was scheduled for the first AFXL CAIRS implantation in Italy in his right eye. Preop uncorrected distance visual acuity (UCVA) was 0.1 decimal equivalents (d. eq), and best spectacles-corrected visual acuity was 0.3 d. eq. with 3 D sphere = −7cylinder axis 20°. The Italian pilot study patient received unanimous and joint approval from the Institutional Review Board of the Siena Crosslinking Center, Siena, and the Vampolieri Eye Clinic, Acicastello, Catania, Italy. The patient signed a specific informed consent form.

This patient was selected for implantation of a single ACXL CAIRS in the right eye according to the MS39 integrated nomogram [16,17,18,19]. This was based on the ICRS Jose F. Alfonso studies [16,17,18,19], which were adapted by the authors’ personalized nomogram based on the direction of the vector axis in the direction of the apex of the cone, according to the preimplant tomographic parameters of the recipient. The first case (single ring) was automatically selected by the MS39 software like “Cone 3B” according to the Jose F. Alfonso nomogram [16,17,18,19] modified by the authors, where the coma axis does not match the astigmatism axis. Primary criteria for implant selection were the coma magnitude and axis, the vectorial direction of the KC apex and the mean pupil power (MPP). The 350 µm (Class 1 MZ CAIRS nomogram) single ring was implanted at 5.5 mm from the corneal vertex. The relative crosslinking protocol was adapted by the authors based on CAIRS thickness according to M nomogram ACXL [20]. The AFXL CAIRS MZ (Mazzotta–Zagari) nomogram for ACXL CAIRSs is displayed in Table 4.

The segment was prepared using an all-femtosecond laser-cut procedure from a donor cornea mounted on an artificial anterior chamber whose parameters are displayed in Table 5.

AFXL CAIRS implantations were performed under topical anesthesia with benoxinate hydrochloride 4 mg/mL eye drops. The second patient (double CAIRS) also underwent reinforcement sub-Tenonian anesthesia with 1.5 cc of lidocaine in a 20 mg/mL injection. The IntraLase™ femtosecond laser (Johnson & Johnson, NJ, USA) was used to create the recipient pockets with a 6 mm optical zone, 2 mm size incision and 2 access cuts for the tunnels. In the first patient, a single pocket was created along the implant axis with a 180° arch to optimize positioning, while in the second case a 360° pocket was created.

**Figure 6:**
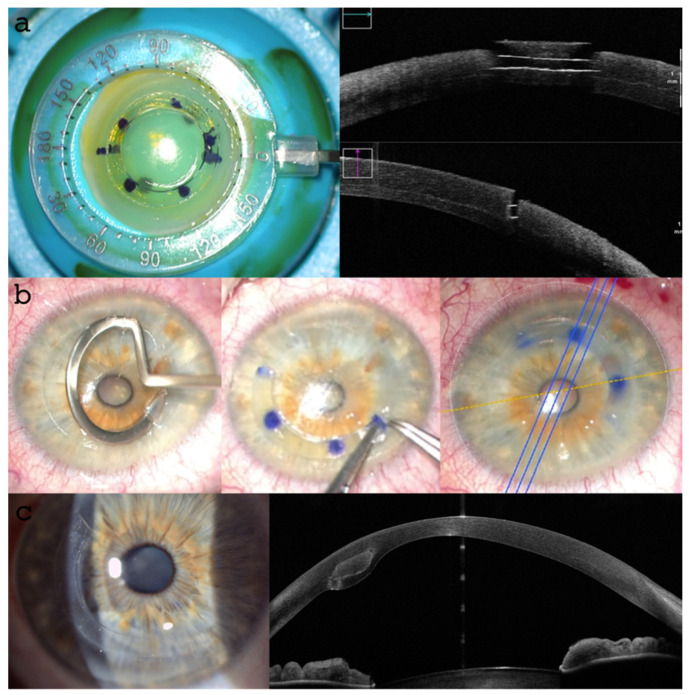
Surgical steps: Preoperative anterior segment (AS) high-resolution OCT highlighted the regular cuts with pre-set angles in the crosslinked tissue (**a**). The surgical technique involved a recipient tunnel formation, segment insertion with a “push-and-pull technique” and the geometric alignment along the major coma aberration axis (**b**). Early postoperative follow-up slit lamp and AS-OCT and high-resolution (HR) images confirmed the correct position of the ACXL CAIRS at the predetermined depth tomographic location and thickness (**c**).

The recipient pocket was opened and conformed with an E. Janach srl (Como, Italy) dissector (J2810.2A) and the single AFXL CAIRS was inserted in a clockwise direction by a “push-and-pull technique” through the 2 incisions. The ring was pushed into the tunnel with a forceps and pulled from the opposite side with a hook (J2179.6), as shown in Figure 6.

In the second patient, the recipient tunnels were opened in two points clockwise by a modified Janach dissector (J2810.2A). The insertion of the CAIRS in this case was performed using a personalized “wire-assisted push-and-pull technique”, suturing the terminal part of the AFXL CAIRS with an 8-0 Vicryl, knotting the suture end to the modified pocket dissector that was introduced into the recipient pocket and exited through the second service incision. After the ring was introduced by pulling the thread, the suture was cut and the conformer retracted. The alignment of the AFXL CAIRS at the end of the procedures was checked by using image-guided marking (Callisto Eye, Carl Zeiss Meditec AG, Jena, Germany) integrated in the operating microscope (Figure 6b).

At the end of surgery, patients were medicated with mydriatic eye drops plus netilmicin dexamethasone eye drops and dressed with a therapeutic soft contact lens for three days. After contact lens removal, netilmicin dexamethasone eye drop association was continued in a tapered regimen for one month (four eye drops in the first 10 days, three in the following 10 days, and 2 in the last 10 days). The antibiotic and steroid therapy was supported by 0.2% hyaluronic acid-based lubricant eye drops.

**Figure 7:**
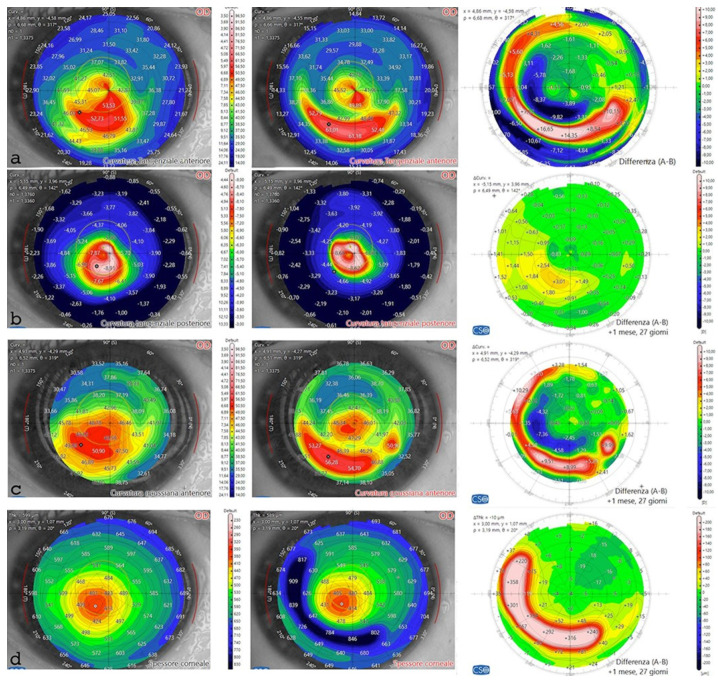
Differential tomography maps after single ACXL CAIRS implant in the first patient in Italy. Anterior tangential map shows flattening of K max from 53.53 D to 49.89 D and topographic hourglass regularization (**a**). Posterior tangential map displays a significant change of +3.01 D postoperatively (**b**). Anterior Gaussian map demonstrates centralization of aberrations with anterior surface flattening of approximately 7.36 D at the most curved point (**c**). Pachymetry differential map indicates increased thickness due to the inserted tissue and stability over the 6-month follow-up (**d**).

**Figure 8:**
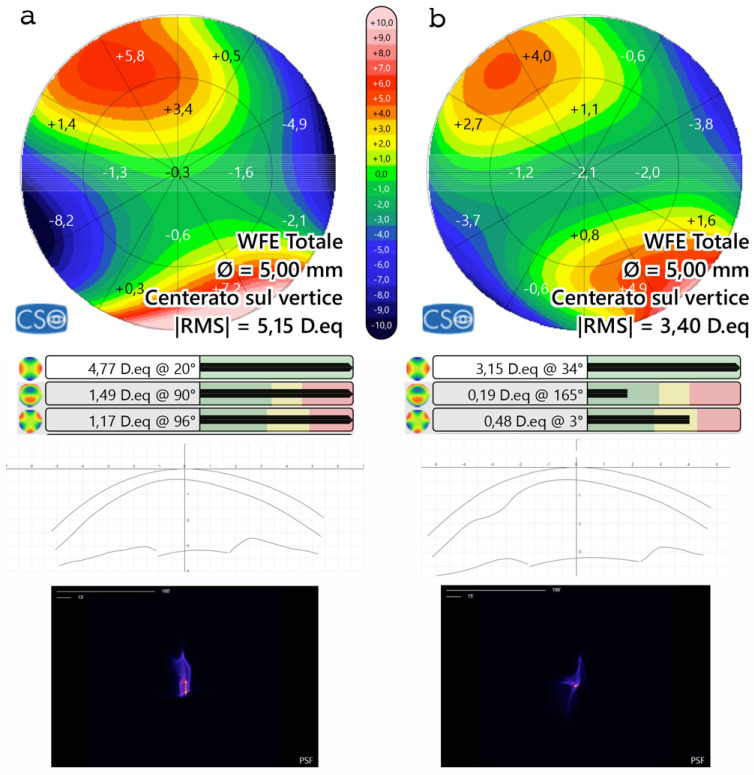
Aberrometric analysis pre- (**a**) and post (**b**) ACXL CAIRS implantation in the first patient in Italy. The comparative images show the improvement in the point spread function (PSF) after ACXL CAIRS implantation and an overall reduction in High-Order Aberrations (HOAs), including coma aberrations and vertical trefoil. Tomographic astigmatism also decreases from 4.77 D to 3.15 D. Additionally, it is noteworthy that the RMS varies from 5.15 D. eq to 3.40 D. eq. These data indicate that ACXL CAIRSs affect not only coma-like aberrations but also other HOAs.

The postoperative analysis revealed the changes in the patient’s corneal profile, Figure 7. Specifically, the maximum Sim K value decreased from 50.00 D to 47.6 D. The corneal wavefront analysis showed a reduction in coma aberration, with values decreasing from 1.49 diopters equivalent (D eq.) axis 90° preoperatively to 0.19 D eq. axis 165° postoperatively (Table 6, Figure 8). A differential anterior tangential map revealed a flattening of K max, with a reduction from 53.53 D to 49.89 D (Figure 7a). The posterior tangential map algorithm showed a significant increase in curvature of +3.01 D (Figure 7b).

The anterior Gaussian differential map showed a flattening of the anterior surface by approximately 7.36 D at the steepest point (Figure 7c). The minimum corneal thickness after ACXL CAIRS tissue addition increased from 362 μm to 378 μm (Figure 7d).

Aberrometric analysis including point spread function (PSF) and coma values showed a reduction in High-Order Aberrations (HOAs) with coma and trefoil improvement (Figure 8). Tomographic astigmatism decreased from 4.77 D to 3.15 D. Additionally, it is noteworthy that the root mean square (RMS) varied from 5.15 D. eq to 3.40 D. eq (Figure 8).

The tissue displacement led to a regularization of the relationship between the anterior and posterior surfaces.

The tissue displacement leads to a regularization of the relationship between the anterior and posterior corneal surfaces. Patient BSCVA passed from 0.3 decimal equivalents (d. eq) with a refraction of −3 sphere = −7cylinder axis 20° to 0.8 d. eq. with a refraction of −2.5 sphere = −1.5 D cylinder axis 20° and uncorrected distance visual acuity (UDVA) improved from 0.1 to 0.5 d. eq.

#### 2.3.2. Patient 2: Double ACXL CAIRS Implant

The second Italian patient scheduled for a double ACXL CAIRS implant was a 46-year-old male patient with stage IV, central symmetric “bow-tie” KC in the left eye. He underwent a penetrating keratoplasty (PKP) in the right eye 15 years prior. The left eye exhibited a UDVA of 0.1 d eq. and a BSCVA of 0.3 d. eq. 3/10 with −6.50 axis 180°. Pre- and postoperative data are displayed in Table 7.

**Figure 9:**
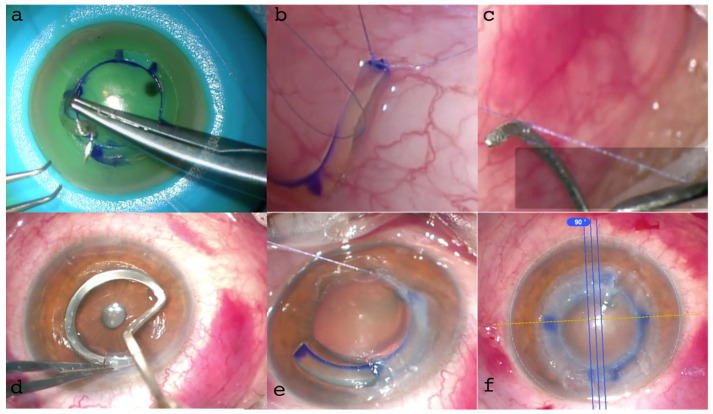
ACXL CAIRS MZ (Mazzotta–Zagari) “wire-assisted push-and-pull technique”. (**a**): passage of the suture thread in the crosslinked segment (**b**): suturing of the ACXL CAIRS with 8-0 Vicryl. (**c**): knotting the suture end to the modified pocket former. (**d**): formation of the corneal pocket. (**e**): introduction of the CAIRS using the personalized Mazzotta–Zagari “wire-assisted push-and-pull technique”. (**f**): alignment of the AFXL CAIRS by image-guided marking (Callisto Eye, Carl Zeiss Meditec AG, Jena, Germany) integrated in the operating microscope alignment of the ACXL CAIRS.

Double CAIRS implant was selected automatically by the MS39 Jose F Alfonso incorporated software [16,17,18,19] as symmetric keratoconus, defined as “bowtie”, Cone Type 5. The nomogram adapted by the authors was a Class 4 KC (Table 4) with a preoperative MPP in the central 3 mm of 54.64 D. Primary criteria for implant selection were coma magnitude, axis of astigmatism, Keratometry and refraction. According to the magnitude of the topographic astigmatism, the nomogram selected the implant of 2 symmetric ring segments. The patient was implanted according to MZ AFXL CAIRS using a personalized wire-assisted (WA) push-and-pull surgical technique, Figure 9. This involved the insertion of two customized trapezoid 160° arcs ACXL CAIRSs with a thickness of 460 µm, a size of 1.5 mm, an 80° inner angle and a 110° outer angle cut with Bowman’s lamina face-up.

**Figure 10:**
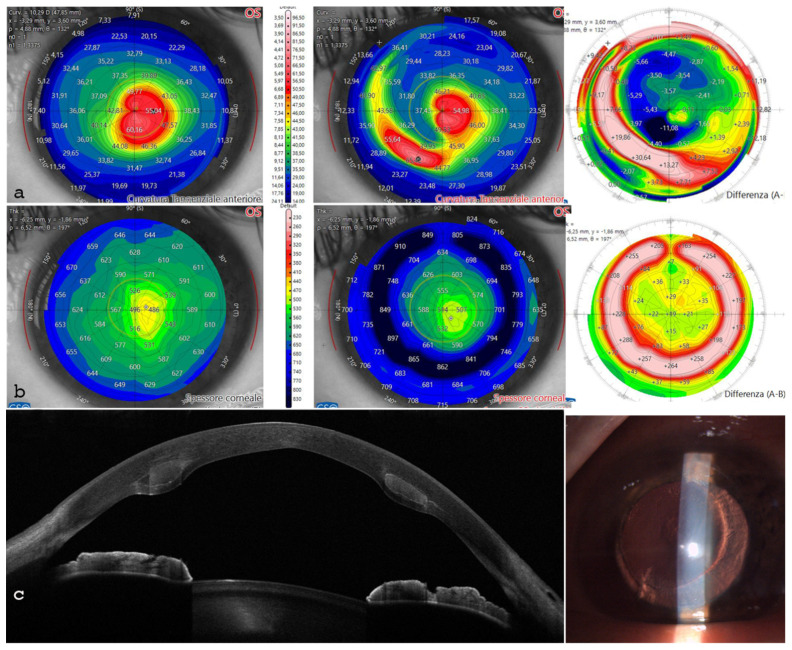
(**a**): the anterior tangential topographic map shows corneal flattening and profile regularization. (**b**): the differential pachymetry map indicates the correct placement of the ACXL CAIRS. (**c**): AS-OCT and slit-lamp images reveal the allogenic segments positioned at 65% planned stromal depth.

The patient achieved a BSCVA of 0.6 d eq. with a refraction of +2 sphere = −5.00 @ 180°. Mean pupil power (MPP) in the central 3 mm optical zone passed from 54.6 to 50.7 D associated with a significant reduction in K max, from 62.4 to 54 D, and accompanied by corneal remodeling and regularization of the tomographic profile. The thinnest point increased from 443 to 486 µm and the Sim K steep values were adjusted from 58.3 to 45.7 D as reported in Table 7 and showed in Figure 10.

## 3. Discussion

CAIRS represents a new frontier in the refractive additive therapy of keratoconus, offering the possibility of reducing complications and the demand for plastic rings and further reducing the need for transplantation as reported in the literature [2,21,22,23].

One of the most important aspects of this technique is the use of human corneal tissues with low endothelial cell counts; since they are not suitable for elective corneal transplantation they can be utilized for this procedure, thus respecting and utilizing the donation [24].

The preparation and insertion of the crosslinked CAIRS using all-femtosecond laser-cut technique allows for better accuracy and customization by eliminating the difficulties associated with the manual technique. In particular, the addition of pre-implantation accelerated crosslinking could, in our opinion, represent a turning point in the so-called AFXL CAIRS technique. This enhancement increases the resistance and viscoelastic consistency of the donor corneal tissue, thus facilitating manipulation and insertion while maintaining the original shape and volume with the planned applanating effect, leading to better efficacy due to their improved strength.

Further, one of the significant benefits of crosslinking the CAIRS lies in its microbicidal activity, which could reduce the chance of post-implantation infectious keratitis.^11^ However, this aspect requires additional study.

The overall data on corneal thickness (both central and at the implant site) and High--Order Aberrations (HOAs) demonstrate the high potential of this additive technique in KC refractive non-replacement therapy, both in the preclinical ex vivo study and in the first two cases performed in Italy. AFXL CAIRSs induce a centripetal tissue shift, similar to a tissue wave, capable of moving over 10 diopters of corneal curvature. Aberrometric analysis including point spread of the AFXL CAIRS tissue displacement leads to a regularization of the relationship between the anterior and posterior corneal surfaces, improving patients’ overall quality of vision (QoV) and BSCVA, decreasing maximum corneal curvature (flattening the anterior curvature), and reducing mean pupil power and astigmatism magnitude. The minimum corneal thickness of the corneas after AFXL CAIRS implantation increased both in ex vivo study and in vivo reports, with +16 µm in the single CAIRS and +43 µm in the double CAIRS implant, respectively (+29 µm on average). No complications or adverse events were reported during the follow-up.

As for the clinical results of the pilot study in Italy with CAIRSs and the first international experience with the use of preimplant ACXL, we firmly believe this is a milestone and the crosslinking of allogenic rings is an added value to be included in the surgical technique in all cases for the purposes of standardization. It would further help mimic the insertion of synthetic segments, therefore making it easier for transitioning surgeons. The remodeling capacity of CAIRS on the shape of irregular corneas, combined with the possibility of implanting the allogenic rings in corneas thinner than 400 µm and with maximum curvatures greater than 55 D, up to 75 D, as reported in the literature [1,2], significantly broadens the indications of non-replacement therapy for keratoconus. This includes more advanced stages that have historically been reserved for deep anterior lamellar keratoplasty (DALK), or at least rigid gas permeable contact lenses (RGP CLs). The limitations of the pilot study are seen in the small sample and the short follow-up lacking blinding and controls. However, the recent literature and these early results demonstrate the possibility of reshaping the cornea even in more advanced KC cases where excimer laser-guided corneal reshaping and synthetic intrastromal rings are not applicable.

## 4. Conclusions

The addition of crosslinking to the CAIRS technique improved the rigidity and the viscoelastic consistency of the corneal allogenic rings while maintaining their dimensions and volume. This facilitated the insertion into the recipient’s tunnels and consequently flattened the learning curve of the technique. Crosslinking the segments in our experience enhanced manipulation and accurate insertion into the tunnels, significantly improving the surgical maneuver. The process did not alter the thickness and volume of the segment compared to the non-crosslinked, dehydrated segments. Additionally, the yellowish coloration of the crosslinked segment facilitated the intraoperative visibility and correct positioning. The possibility of increasing corneal thickness and decreasing warping and aberrations, as this pilot study has demonstrated by showing an increase in corneal thickness in all cases of implantation and a remodeling of the corneal profile with reduction in corneal curvature, High-Order Aberrations and improvement in minimum corneal thickness, can pave the way for future refractive refinement using ray-tracing-based excimer laser corneal remodeling [20].

### 4.1. What Was Known

- Corneal allogenic intrastromal ring segments (CAIRSs) represent a new therapeutic, corneal transplant-sparing approach for the treatment of keratoconus.
- Corneal allogenic intrastromal ring segments (CAIRSs) can be an alternative to synthetic ring segments, eliminating their complications and being implantable in more advanced cases.
- All-femtosecond laser-cut crosslinked corneal allogenic intracorneal ring segments (AF-CAIRSs) facilitated customization and improved clinical results in corneal reshaping.

### 4.2. What This Paper Adds

- All-femtosecond laser-cut crosslinked corneal allogenic intrastromal ring segments (AFXL CAIRSs) further facilitate the manipulation and insertion of the segments, increasing their resistance and viscoelastic consistency, thereby flattening the learning curve of this technique.
- Crosslinking of CAIRSs can be an added value in the surgical technique, aiding in its future standardization.

## Figures and Tables

**Table 1:** Phase 1 ex vivo study: AFXL CAIRS characteristics. Legend: AFXL CAIRS: crosslinked all-femtosecond laser-cut corneal allogenic ring segment; HPMC: hydroxypropyl methylcellulose (HPMC); UV-A: ultraviolet A rays.

AFXL CAIRS	Thickness (µm)	Diameter (mm)	Arch Length (°)	Total Crosslinking Time (min)	Riboflavin 0.1% HPMC Soaking Time (min)	UV-A Irradiation (min)	Depth of Implant (%)	Recipient Tunnel Diameter (mm)	Donor Central Corneal Thickness (µm)
1	350	1.5	160	8	5	3	50	2	600
2	350	1.5	160	10	5	5	60	2	530
3	350	1.5	160	15	5	10	70	2	517

**Table 2:** Changes in corneal thickness, maximum corneal curvature (K max) and High-Order Aberration (HOA) values following ACXL CAIRS implantation. Legend: High-Order Aberrations (HOAs), diopters (Ds), recipient corneas (Rs).

Recipient Corneas	Average Central Thickness pre µm	Average Central Thickness Post µm	Thickness in the Site of Implant pre µm	Thickness in the Site of Implant Post µm	K max pre D	K max post D	HOA pre D 6 mm	HOA post D 6 mm
R1	405	405	500	800	50.43	59.38	0.31	2.51
R2	459	553	456	806	51.65	69.76	0.47	3.49
R3	462	473	463	813	53.58	68.3	0.99	7.30

**Table 3:** Average maximum corneal curvature pre- and post ACXL-CAIRS implantation. The average and standard deviation (SD) of K max pre- and post-implantation showed a change in the corneal shape through a centripetal wave movement of corneal tissue of 13.93 D (±5.62 SD) in K max.

K max pre (D)	Pre (D)	Post (D)	ΔK (D)
K max (average)	51.89	65.81	13.93
K max (standard deviation)	1.59	5.62	

**Table 4:** MZ (Mazzotta–Zagari) CAIRS personalized nomogram used for AFXL CAIRSS. Legend: CLASS: ectasia classification; MPP Ds: mean pupil power diopters; CAIRS T: CAIRS thickness; A/P ELr: Anterio/Posterior Elevation ratio; IA CUT: Internal Angle femtosecond laser cut; EA CUT: External Angle femtosecond laser cut; RS min: Riboflavin Soaking minutes; CXLF: Crosslinking Fluence; UVP/T: UVA power/time of exposure minutes.

CLASS	MPP Ds	CAIRS T μm	AP ELr μm	ARC Degrees	IA CUT Degrees	EA CUT Degrees	RS min	CXLF Joules	UVP/T mW/mins
1	≥45	=350	<20	160 ± 20	60–90	90–120	5	5.4	30/6
2	46–48	>350–400	>20–30	160 ± 20	60–90	90–120	7	5.4	18/10
3	49–53	>400–450	>30–40	160 ± 20	60–90	90–120	8	5.4	15/12
4	>53	>450	>40	160 ± 20	60–90	90–120	10	5.4	9/10

**Table 5:** AFXL-CAIRS all-femtosecond laser-cut parameters.

Parameters	Donor	Recipient
Depth in cornea (µm)	400	382 (65%)
Diameter (mm)	9.2	160° (arc)
Inner diameter (mm)	6.0	5.8
Inner angle cut (°)	70	-
Outer diameter (mm)	7.5	7.8
Outer angle cut (°)	110	-
Energy (micro J)	1.50	1.50

**Table 6:** Patient 1, single AFXL CAIRS pre- and postoperative parameters.

Parameter	Preoperative	Postoperative
MPP 3 mm	45.32 D	44.30 D
SimK (K steep)	53.53 D	49.89 D
Astigmatism	−7.25 D@25°	−3.71 D@30°
Coma Aberrations	1.49 D eq. @90	0.19 D @165°
Thinnest Point (µm)	362	378

**Table 7:** Patient 2, double AFXL CAIRS implant pre- and postoperative data.

Parameter	Preoperative	Postoperative
MMP 3 mm	54.64 D	50.73 D
SimK (K steep)	58.30 D	45.75 D
Astigmatism	−7.25 D axis 25°	−4.75 D axis 30°
K Max	62.41 D	54.01 D
Coma Aberrations	1.49 D eq. Axis 90	D axis 165°
Thinnest Point (µm)	443	486

## Data Availability

The original contributions presented in the study are included in the article, further inquiries can be directed to the corresponding authors.
